# A New Methodology for Multiscale Myocardial Deformation and Strain Analysis Based on Tagging MRI

**DOI:** 10.1155/2010/341242

**Published:** 2010-03-01

**Authors:** Luc Florack, Hans van Assen

**Affiliations:** ^1^Department of Mathematics & Computer Science, Eindhoven University of Technology, P.O. Box 513, 5600 MB Eindhoven, The Netherlands; ^2^Department of Biomedical Engineering, Eindhoven University of Technology, P.O. Box 513, 5600 MB Eindhoven, The Netherlands

## Abstract

Myocardial deformation and strain can be investigated using suitably encoded cine MRI that admits disambiguation of material motion. Practical limitations currently restrict the analysis to in-plane motion in cross-sections of the heart (2D + time), but the proposed method readily generalizes to 3D + time. We propose a new, promising methodology, which departs from a multiscale algorithm that exploits local scale selection so as to obtain a robust estimate for the velocity gradient tensor field. Time evolution of the deformation tensor is governed by a first-order ordinary differential equation, which is completely determined by this velocity gradient tensor field. We solve this matrix-ODE analytically and present results obtained from healthy volunteers as well as from patient data. The proposed method requires only off-the-shelf algorithms and is readily applicable to planar or volumetric tagging MRI sampled on arbitrary coordinate grids.

## 1. Introduction

**Figure 1:**
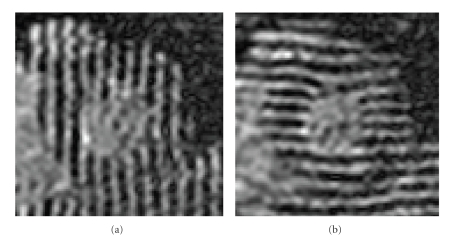
Vertical (a) and horizontal (b) SPAMM encoding in a basal short-axis slice at approximately mid-systole for a patient.

Cine MRI, combined with (C)SPAMM ((C)SPAMM = (Complementary) SPAtial Modulation of Magnetization) encoding technology [1–4], admits disambiguation of local tissue motion, thus enabling the extraction of myocardial deformation and strain [5], which are known to correlate with cardiac pathologies. In particular, Götte et al. found that strain is more accurate than geometry in discriminating dysfunctional from functional myocardium [6]. Deformation and strain can be operationalized in various ways, either without explicit a priori regularization, or through exploitation of sparse constraints combined with interpolation and/or regularization [5, 7–33]. Possible encodings are (DENSE) Displacement ENcoding with Stimulated Echoes, cf. Aletras et al. [2), and (HARP) HARmonic Phase, cf. Osman et al. [20, 21]. Tagging-based methods using HARP technology form our point of departure, compare with Figure 1 for an illustration.

Given a dense motion field within the myocardium, our aim is to devise an operational procedure for direct extraction of myocardial deformation and strain. By “direct” we mean that we seek to obviate sophisticated preprocessing steps, such as segmentation of, or interpolation between tag lines, and finite element methods explicitly coupled to the tagging pattern. Although such sophisticated “indirect” procedures exist and have been proven powerful, they require specific algorithmics that is neither trivially implemented nor readily available. Instead we aim for a multiscale, optimally conditioned, intrinsically parallelizable, linear algorithm for obtaining deformation (and thus strain) in analytically closed form. Optimal conditioning is achieved by exploitation of the scale degree of freedom in the definition of spatiotemporal differential image structure. In addition we aim to minimize the number of extrinsic control parameters. We believe that the parsimony of our method facilitates applicability and optimization, since only off-the-shelf algorithms (linear filtering and inversion of linear systems) are needed in our computation of myocardial deformation and strain.

Our approach is as follows. To begin with, the *velocity gradient tensor field*, which is the prerequisite for the model we present in this paper, is obtained using the multiscale motion extraction algorithm for scalar image sequences proposed by Florack et al. [12]. This algorithm is adapted to the situation of tagging MRI data [10, 13]. Slick encoding and/or linear filtering yields an *n*-tuple of independent scalar phase images of some fixed spatiotemporal region of interest, in which *n* is the dimension of space (*n* = 2,3, as may be the case). The intrinsic, *n*-fold underdetermined “optic flow constraint equation” can, by construction, be applied to each of these *n* phase images, yielding an unambiguous system of equations for the underlying dense motion field to any desired differential order, and obviating Tikhonov regularization (which would bring in at least one additional control parameter) as a disambiguating prior (as is typically the case in optic flow applications due to the aperture problem). It has been verified that the first-order scheme indeed produces a plausible, dense, and robust velocity gradient tensor field within the myocardium. Details of its construction as well as the underlying automatic scale selection mechanism can be found in the cited literature (for an example of automatic scale selection, cf. also Niessen et al. [34]). In view of the cited work we will de-emphasize multiscale motion extraction and concentrate on subsequent deformation and strain analysis.

In Section 2 we outline in detail how to arrive at a closed-form analytical solution for the *deformation tensor field* (Section 2.1) and hence the *strain tensor field* (Section 2.2), given the velocity gradient tensor field. The novelty of this approach is that we circumvent numerical approximations in all intermediate steps, and introduce numerics only at the ultimate stage where we sample the resulting analytical tensor field expressions. Besides avoiding in this way numerical errors that may be difficult to quantify, this procedure has the advantage of being mathematically transparent, computationally trivial, and intrinsically parallellizable. (These properties, in fact, also hold for the multiscale motion extraction algorithm used to provide the input velocity gradient tensor field, loc. cit.) Some experimental results are given in Section 3 to demonstrate the feasibility of our approach.

Section 4 concludes our work with a summary and briefly sketches work in progress. 

Data acquisition details for the scans used in this paper are given in the appendix.

## 2. Theory

### 2.1. Deformation

The velocity gradient tensor, with components *L*_*β*_^*α*^ relative to a coordinate frame, relates the rate of change of a momentary infinitesimal line element $d{\dot{x}}^{\alpha }$ to the line element *d* *x* ^*β*^ itself. (One can either view *d* *x* ^*α*^ as the *α*-component of an “infinitesimal vector” attached to a fiducial material point in Euclidean space and representing a directed material line element, that is, an “infinitesimal” number—or as the *α*th element of a local covector frame, depending on engineering or mathematical mind set.) From $d{\dot{x}}^{\alpha }=dv^{\alpha }$ it follows, using the chain rule, that

(1) $$\begin{array}{c} d{\dot{x}}^{\alpha }=L_{\beta }^{\alpha }\;dx^{\beta }\;\text{with}\;\;L_{\beta }^{\alpha }=\frac{\partial v^{\alpha }}{\partial x^{\beta }}\left(\alpha ,\beta =1,\ldots ,n\right). \end{array}$$

(The Einstein summation convention applied here will be used henceforth.) The numbers *L*_*β*_^*α*^ constitute a matrix **L** with row and column index *α*, *β*. To get an estimate of this tensor field we have applied the algorithm of Florack et al. and van Assen et al., solving a linear system of algebraic equations enforcing optimal conditioning through scale selection. The reader is referred to the literature for details [10, 12, 13].

Tissue deformation can be described by a map *f* : *M* → *N* : *x*(*X*, *t*_0_) ↦ *x*(*X*, *t*), in which *x*(*X*, *t*) denotes the spatial position of a material point at time *t*, with reference position *X* = *x*(*X*, *t*_0_) at time *t*_0_ (Lagrange picture). Domain *M* and codomain *N* are copies of the deformable tissue medium (a subset of ℝ^*n*^) at times *t*_0_, respectively, *t* ≥ *t*_0_. We fix *t*_0_ so as to correspond to end-diastole. (During the diastolic phase the heart relaxes and is refilled with blood. It is followed by the systolic phase, in which the heart contracts. It is this phase that traditionally receives most attention by cardiologists.)

The associated differential map,

(2) $$\begin{array}{c} f_{*}\equiv df:TM_{X}\to TN_{x}:v\mapsto f_{*}\left(v\right)=v^{i}\;F_{i}^{\alpha }\;f_{\alpha }, \end{array}$$

relative to a basis {**f**_*α*_} of *T* *N*_*x*_, provides a local linearization of tissue deformation, that is, the deformation tensor. Here *T* *M*_*X*_ is the tangent space of *M* at the fiducial point *X* = *x*(*X*, *t*_0_), and *T* *N*_*x*_ that of *N* at the *f*-mapped point *x* = *x*(*X*, *t*). The matrix **F**(*t*, *t*_0_) of this linear map relative to the local coordinate charts {*x* ^*α*^, *N*} and {*X* ^*i*^, *M*} is given by the Jacobian

(3) $$\begin{array}{c} F_{i}^{\alpha }=\frac{\partial x^{\alpha }}{\partial X^{i}}. \end{array}$$

By virtue of the chain rule, the relation between deformation and velocity gradient tensors, (1) and (3) is given by the first-order ODE [35]

(4) $$\begin{array}{c} \dot{F}=L\;F, \end{array}$$

subject to an initial condition, namely, **F**(*t* = *t*_0_, *t*_0_) = **I**. Equivalently,

(5) $$\begin{array}{c} F\left(t,t_{0}\right)=I+\int_{t_{0}}^{t}\;L\left(s\right)\;F\left(s,t_{0}\right)\;ds. \end{array}$$

Only for stationary flows, that is, **L**(*t*) = **L**_0_ pointwise constant, this initial value problem admits a trivial solution **F**(*t*, *t*_0_) = exp ((*t* − *t*_0_)**L**_0_). (For the sake of simplicity the spatial dependency of all field entities is suppressed in the notation.) However, we do not have stationarity, and so we must proceed more carefully.

 Equation (5) induces an expansion known as the *matricant*, compare with Gantmacher [35]:

(6) $$\begin{array}{c} F\left(t,t_{0}\right)=I+\int_{t_{0}}^{t}L\left(\tau \right)d\tau +\int_{t_{0}}^{t}L\left(\tau \right)\int_{t_{0}}^{\tau }L\left(\sigma \right)d\sigma \;d\tau +\cdots . \end{array}$$

The matricant has the following property:

(7) $$\begin{array}{c} F\left(t,t_{0}\right)=F\left(t,t_{1}\right)F\left(t_{1},t_{0}\right)\;\left(t_{0}<t_{1}<t\right). \end{array}$$

It follows that, if we split the interval [*t*_0_, *t*] into *n* parts by using intermediate points *t*_1_,…, *t*_*n*−1_ separated by Δ*t*_*k*_ = *t*_*k*_ − *t*_*k*−1_ (*k* = 1,…, *n*, with *t*_*n*_ = *t*),

(8) $$\begin{array}{cc} F\left(t,t_{0}\right)=F\left(t,t_{n-1}\right)\cdots F\left(t_{1},t_{0}\right), & \\ \text{with}\;\;t_{0}<t_{1}<\cdots <t_{n-1}<t_{n}=t. & \end{array}$$

For an infinitesimally narrow time interval [*t*_*k*−1_, *t*_*k*_] we have by approximation

(9) $$\begin{array}{c} F\left(t_{k},t_{k-1}\right)=I+L\left(t_{k}^{*}\right)\;\Delta t_{k}+\text{higher}\;\;\text{order}\;\;\text{terms}\;\;\text{in}\;\;\Delta t_{k}, \end{array}$$

in which *t*_k_* is any point satisfying *t*_*k*−1_ ≤ *t*_k_* ≤ *t*_*k*_. Equations (8) and (9) give rise to a representation in terms of a so-called *multiplicative integral* [35]:

(10) $$\begin{array}{cc} F\left(t,t_{0}\right) & =\sim \int_{t_{0}}^{t}\left(I+L\left(\tau \right)d\tau \right)\\ & \overset{\mathrm{def}}{=}\underset{\Delta t_{k}\to 0}{\lim\;}\left(I+L\left(t_{n}^{*}\right)\Delta t_{n}\right)\cdots \left(I+L\left(t_{1}^{*}\right)\Delta t_{1}\right). \end{array}$$

One recognizes the multiplicative counterpart of the Riemann sum approximation for ordinary (additive) integrals. One can show that this is identical to

(11) $$\begin{array}{cc} F\left(t,t_{0}\right) & \overset{\left[35\right]}{=}\sim \int_{t_{0}}^{t}\exp\;\left(L\left(\tau \right)\;d\tau \right)\\ & \overset{\mathrm{def}}{=}\underset{\Delta t_{k}\to 0}{\lim\;}\exp\;\left(L\left(t_{n}^{*}\right)\Delta t_{n}\right)\cdots \exp\;\left(L\left(t_{1}^{*}\right)\;\Delta t_{1}\right). \end{array}$$

Several properties of the deformation tensor are manifested in this representation. For instance, for square matrices **A**, **B**, one has

- det  **A** **B** = det  **A**det  **B**,
- det  (**I** + *ϵ* **A**) = 1 + *ϵ*tr   **A** + *𝒪*(*ϵ* ^2^),
- det  exp  **A** = exp  tr   **A**.

Consequently,

(12) $$\begin{array}{c} \det\;F\left(t,t_{0}\right)=\sim \int_{t_{0}}^{t}\left(1+\mathrm{tr}\;\;L\left(\tau \right)d\tau \right)=\sim \int_{t_{0}}^{t}\exp\;\left(\mathrm{tr}\;\;L\left(\tau \right)d\tau \right). \end{array}$$

In particular, a divergence free velocity field (div   **v** = tr   **L** = 0) preserves volumes: det  **F**(*t*, *t*_0_) = 1. Furthermore, exp  **A**exp  **B** = exp  (**A** + **B**) if [**A**, **B**] = 0, whence for a stationary velocity field one obtains **F**(*t*, *t*_0_) = exp  ((*t* − *t*_0_)**L**_0_), as we already noticed. Finally, the multiplicative integral suggests a straightforward numerical approximation, namely by using either (10) or (11) without limiting procedure. In this case the two representations are of course no longer identical. For computational efficiency we have chosen the former, with Δ*t*_*k*_ = Δ*t* corresponding to the (constant) frame interval of our tagging MRI sequence, and *t*_k_* = *k*Δ*t*.

### 2.2. Strain

On the basis of the differential map *f*_∗_, (2), and its transpose *f*_∗_^T^, one defines an intrinsic mapping on *T* *M*_*X*_, known as the *Lagrangian strain tensor* [36],

(13) $$\begin{array}{cc} & E\equiv \frac{1}{2}\left(f_{*}^{\text{T}}\circ f_{*}-\text{i}{\text{d}}_{TM_{X}}\right):TM_{X}\\ & \to TM_{X}:v\\ & \mapsto E\left(v\right)=v^{i}E_{i}^{j}e_{j}, \end{array}$$

relative to a basis {**e**_*j*_} of *T* *M*_*X*_, with mixed tensor components

(14) $$\begin{array}{c} E_{i}^{j}=\frac{1}{2}\left(g^{\ell j}F_{\ell }^{\alpha }h_{\alpha \beta }F_{i}^{\beta }-\delta_{i}^{j}\right). \end{array}$$

Here *g* ^*ij*^ are the components of the dual Euclidean metric tensor in domain coordinates {*X* ^*i*^, *M*}, and *h*_*αβ*_ are those pertaining to codomain coordinates {*x* ^*α*^, *N*}. *E* vanishes identically if *f*_∗_ is an isometry, thus *E* captures genuinely nonrigid deformations.

The general coordinate convention, see (14), which admits different bases for deformed and undeformed configurations, is instructive. Although one would normally prefer identical bases, let us consider the case in which the metric components *h*_*αβ*_ are those induced from *g*_*ij*_ by the deformation map itself (carry-along). That is, we assume that the coordinate frame for the deformed configuration is just the deformed coordinate frame of the reference configuration. One then expects the Lagrangian strain tensor to be nullified, because everything, including the local reference frames, is intrinsically deformed in a consistent manner. Indeed, if we write the infinitesimal material line element as

(15) $$\begin{array}{c} ds^{2}=g_{ij}dX^{i}dX^{j}=g_{ij}\frac{\partial X^{i}}{\partial x^{\alpha }}\frac{\partial X^{j}}{\partial x^{\beta }}dx^{\alpha }dx^{\beta }\equiv h_{\alpha \beta }dx^{\alpha }dx^{\beta }, \end{array}$$

we recognize the deformation tensor, see (3), and it follows that

(16) $$\begin{array}{c} g_{ij}=h_{\alpha \beta }F_{i}^{\alpha }F_{j}^{\beta }. \end{array}$$

As anticipated for the coordinate frames employed, (14) indeed reduces to

(17) $$\begin{array}{c} E_{i}^{j}=\frac{1}{2}\left(g^{\ell j}\underset{\left(16\right)}{\underset{︸}{h_{\alpha \beta }F_{\ell }^{\alpha }F_{i}^{\beta }}}-\delta_{i}^{j}\right)=\frac{1}{2}\left(g^{\ell j}g_{\ell i}-\delta_{i}^{j}\right)=0. \end{array}$$

In practice one of course carries out computations relative to fixed (deformation independent) coordinate frames for undeformed and deformed configurations, but there is no reason to assume input and output frames to coincide a priori, nor to restrict oneself to Cartesian coordinate frames. Equation (14) permits us to use whatever bases we may consider convenient. This may be beneficial in certain situations, for example, those that suggest spherical, cylindrical or other coordinate systems depending on the configuration of acquisition data, compare with the phantom study by Young et al. [37], and the desired output representation.

Below we consider a single Cartesian coordinate system for both domain and codomain. (In such a system the representations of the various metric tensors, *g*_*ij*_ and *h*_*αβ*_ and their duals, *g* ^*ij*^ and *h* ^*αβ*^, all simplify to identity matrices.) Our analysis is confined to a single short-axis plane, whence *n* = 2, that is, we only account for in-plane motion components. The theory trivially generalizes to *n* = 3 and non-Cartesian coordinate grids.

## 3. Experiment

**Figure 2:**
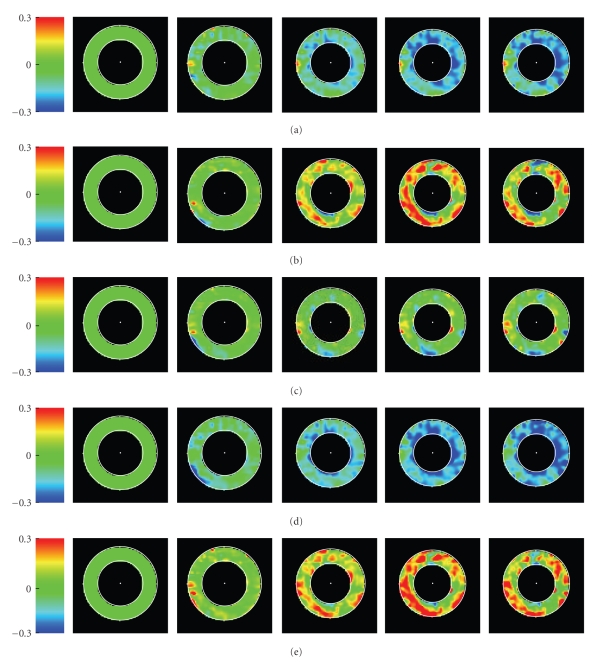
Color-coded strain fields in short axis mid-slice cross-sections, time frames 1,6, 11,16,21 (a full heart cycle subtends 42 frames), regularized through Gaussian convolution with spatial scale *σ* = 1.0, for one healthy volunteer. First row: Circumferential strain *E*_cc_. Second row: Radial strain *E*_rr_. Third row: Shear strain *E*_cr_. Fourth row: Minimal strain eigenvalue *E*_min _. Fifth row: Maximal strain eigenvalue *E*_max _.

Figure 2 illustrates various scalar fields extracted from the strain tensor field, (13) and (14), by contraction with a pair of local unit vectors, namely radial (*r* = radial) and azimuthal (*c* = circumferential) basis vectors of the polar coordinate system centered at the midpoint of the region of interest, and those defining the strain tensor's eigensystem. If *u*, *v* ∈ *T* *M*_*X*_ are two such unit vectors, then the local scalar quantity derived from the strain tensor is given by the inner product (*u*, *E*(*v*)) = (*E*(*u*), *v*) = *g*_*jk*_ *u* ^*k*^ *E*_*i*_^*j*^ *v* ^*i*^. Tensor components are evaluated in Cartesian coordinates.

**Figure 3:**
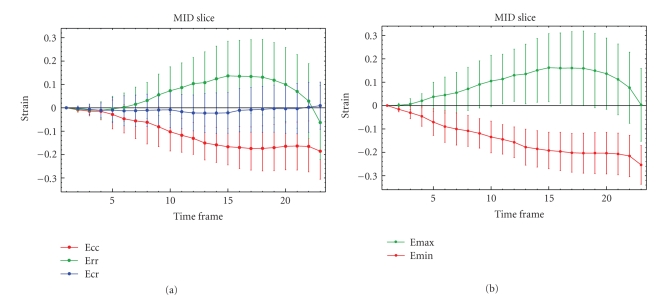
Temporal evolution of scalar strain quantities over the first half of the heart cycle (i.e., mostly systolic) in mid-slice cross-section. Error bars indicate standard deviations over the spatial ROI in each time frame, thus capturing all sources of variation due to (primarily) actual spatial variability, noise, and numerics. Legends explain the various graphs. Notice the strong correlation between the extrinsic (polar system related) and instrinsic (eigensystem related) strains. (In the eigensystem, shear strain vanishes identically.)

**Figure 4:**
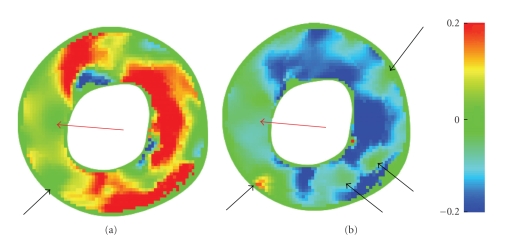
Radial (a) and circumferential (b) colour-coded strains in a basal short-axis slice at approximately end-systole for a patient. Red arrows on the left indicate regions where the respective strain values deviate from normal. These regions were confirmed to be static by a cardiologist. The patient's anamnesis mentions small infarcts on either side of these regions. Their locations, indicated by black arrows, are highlighted in late-enhancement MRI.

Figure 3 illustrates temporal evolutions of these quantities spatially averaged over the respective regions of interest, together with their standard deviations. (Thus one should *not* confuse standard deviations with uncertainties in this plot.) Table 1 shows statistics for three healthy volunteers. Figure 4 shows that strain fields have the potential to detect local pathology.

## 4. Conclusion and Future Work

We have proposed a novel, simple and robust linear model for extracting myocardial deformation and strain. Material motion and gradient velocity are determined by a multiscale linear system of algebraic equations for the phase images extracted from the tagging MRI data. These phase images are scalars, not densities, and are not hampered by tag fading due to *T*_1_ relaxation.

We have analytically solved the linear matrix-ODE governing myocardial deformation. By discretizing the closed-form solution we have subsequently solved for the induced Lagrangian strain tensor field, yielding results that are typical for healthy volunteers, compare with Garot et al. [14], and atypical for a patient with a medical history of small infarcts on either side of a deviatory region and confirmed by late-enhancement MRI. This demonstrates the feasibility of our method. An advantage of our method is that only off-the-shelf algorithms are needed. This serves clarity, facilitates optimization, and enables implementation on dedicated hardware. The method is applicable in any spatial dimension, does not require conversion to a Cartesian sampling grid, is optimally conditioned due to local scale selection, and is readily adapted to other modalities such as velocity encoding MRI.

Our quantitative results in this feasibility study demand further evaluation so as to reveal their statistical significance and clinical value. Furthermore, experiments based on carefully controlled synthetic or phantom data will allow us to disclose the physical significance and numerical tolerance of the mathematical framework in terms of ground truth deformation properties. Moreover, by virtue of the transparent mathematical theory, it is also possible to assess tolerances on the basis of theoretical error propagation models [38], which can then be compared to those established experimentally for synthetic and phantom data. Consistency will greatly increase the confidence of the method for in vivo studies. Finally, although the local scale selection criterion exploited in our approach guarantees optimal conditioning, it does not necessarily yield optimal results due to the intrinsically parallel and thus spatiotemporally uncorrelated nature of the selected neighbouring scales. The effect of spatiotemporal regularization on the pattern of locally selected scales needs to be investigated. Future investigation will be needed to pursue all these recommendations.

## Figures and Tables

**Table 1:** Spatial average and standard deviation of strains from three healthy volunteers (labels I, II, III) over base, mid, and apex short-axis cross-sectional ROIs at that time frame at which *E*_*max*_ has attained its maximum in mid-slice (*t*_*I*_ = 16, *t*_*II*_ = 15, *t*_*III*_ = 14). Rows correspond to, from top to bottom, *E*_cc_, *E*_rr_, *E*_cr_, *E*_max _, and *E*_min _.

Base			MID			APEX		
I	II	III	I	II	III	I	II	III
−0.16 ± 0.13	−0.17 ± 0.11	−0.19 ± 0.11	−0.19 ± 0.09	−0.17 ± 0.08	−0.15 ± 0.09	−0.16 ± 0.16	−0.20 ± 0.09	−0.19 ± 0.13
0.20 ± 0.25	0.16 ± 0.21	0.14 ± 0.25	0.04 ± 0.20	0.14 ± 0.15	0.03 ± 0.15	0.07 ± 0.23	0.20 ± 0.23	−0.02 ± 0.18
0.02 ± 0.15	−0.04 ± 0.12	−0.07 ± 0.13	0.01 ± 0.07	−0.02 ± 0.09	−0.06 ± 0.11	0.07 ± 0.14	−0.03 ± 0.11	−0.05 ± 0.12
0.25 ± 0.22	0.19 ± 0.20	0.18 ± 0.23	0.07 ± 0.17	0.16 ± 0.15	0.09 ± 0.16	0.15 ± 0.21	0.22 ± 0.22	0.05 ± 0.19
−0.22 ± 0.11	−0.21 ± 0.09	−0.24 ± 0.09	−0.23 ± 0.09	−0.19 ± 0.08	−0.21 ± 0.07	−0.26 ± 0.12	−0.23 ± 0.09	−0.27 ± 0.09

## Appendix

### Data Acquisition Details

The short-axis MR tagging image data used in this study were acquired with a Philips Intera 1.5 T scanner from three volunteers (Table 1) and a patient in basal, midventricular, and apical slices. The patient (Figure 4) had a history of severe stenoses, and small infarction areas confirmed with late-enhancement MRI 14 minutes after gadolinium contrast injection. A 2D multishot gradient-echo with Echo Planar Imaging (EPI factor 9) with breath-holding in end-expiration was used. Scan parameters were: TE 4.4 ms, TR 19 ms, flip angle 10°, field-of-view 300 mm, scan matrix 128, acquisition voxel size 2.34 × 2.68 × 8 mm^3^ reconstructed into 1.17 × 1.17 × 8 mm^3^. Tagline spacing was 8 mm.

## References

[1] Axel L, Dougherty L (1989). MR imaging of motion with spatial modulation of magnetization. *Radiology*.

[2] Axel L, Dougherty L (1989). Heart wall motion: improved method for spatial modulation of magnetization for MR imaging. *Radiology*.

[3] Fischer SE, McKinnon GC, Maier SE, Boesiger P (1993). Improved myocardial tagging contrast. *Magnetic Resonance in Medicine*.

[4] Zerhouni EA, Parish DM, Rogers WJ, Yang A, Shapiro EP (1988). Human heart: tagging with MR imaging: a new method for noninvasive assessment of myocardial motion. *Radiology*.

[5] Axel L, Montillo A, Kim D (2005). Tagged magnetic resonance imaging of the heart: a survey. *Medical Image Analysis*.

[6] Götte MJW, van Rossum AC, Twisk JWR, Kuijer JPA, Marcus JT, Visser CA (2001). Quantification of regional contractile function after infarction: strain analysis superior to wall thickening analysis in discriminating infarct from remote myocardium. *Journal of the American College of Cardiology*.

[7] Aletras AH, Balaban RS, Wen H (1999). High-resolution strain analysis of the human heart with fast-DENSE. *Journal of Magnetic Resonance*.

[8] Aletras AH, Ding S, Balaban RS, Wen H (1999). DENSE: displacement encoding with stimulated echoes in cardiac functional MRI. *Journal of Magnetic Resonance*.

[9] Aletras AH, Wen H (2001). Mixed echo train acquisition displacement encoding with stimulated echoes: an optimized DENSE method for in vivo functional imaging of the human heart. *Magnetic Resonance in Medicine*.

[10] van Assen H, Florack L, Suinesiaputra A, Westenberg J, ter Haar Romeny BM, Miller K, Paulsen KD, Young AA, Nielsen PMF Purely evidence based multiscale cardiac tracking using optic flow.

[11] Axel L, Chen T, Manglik T, Frangi AF, Radeva PI, Santos A, Hernandez M Dense myocardium deformation estimation for 2D tagged MRI.

[12] Florack L, Niessen W, Nielsen M (1998). The intrinsic structure of optic flow incorporating measurement duality. *International Journal of Computer Vision*.

[13] Florack L, van Assen H, Suinesiaputra A, Niessen W, Westin C-F, Nielsen M Dense multiscale motion extraction from cardiac cine MR tagging using HARP technology.

[14] Garot J, Bluemke DA, Osman NF (2000). Fast determination of regional myocardial strain fields from tagged cardiac images using harmonic phase MRI. *Circulation*.

[15] Gilson WD, Yang Z, French BA, Epstein FH (2004). Complementary displacement-encoded MRI for contrast-enhanced infarct detection and quantification of myocardial function in mice. *Magnetic Resonance in Medicine*.

[16] Gupta SN, Prince JL, Bizais Y On variable brightness optical flow for tagged MRI.

[17] Haber I, Kikinis R, Westin CF Phase-driven finite element model for spatio-temporal tracking in cardiac tagged MRI.

[18] Kraitchman DL, Sampath S, Castillo E (2003). Quantitative ischemia detection during cardiac magnetic resonance stress testing by use of FastHARP. *Circulation*.

[19] Kuijer JPA, Hofman MBM, Zwanenburg JJM, Marcus JT, Van Rossum AC, Heethaar RM (2006). DENSE and HARP: two views on the same technique of phase-based strain imaging. *Journal of Magnetic Resonance Imaging*.

[20] Osman NF, Kerwin WS, McVeigh ER, Prince JL (1999). Cardiac motion tracking using CINE harmonic phase (HARP) magnetic resonance imaging. *Magnetic Resonance in Medicine*.

[21] Osman NF, McVeigh ER, Prince JL (2000). Imaging heart motion using harmonic phase MRI. *IEEE Transactions on Medical Imaging*.

[22] Ozturk C, Derbyshire JA, McVeigh ER (2003). Estimating motion from MRI data. *Proceedings of the IEEE*.

[23] Pan L, Prince JL, Lima JAC, Osman NF (2005). Fast tracking of cardiac motion using 3D-HARP. *IEEE Transactions on Biomedical Engineering*.

[24] Prince JL, McVeigh ER (1992). Motion estimation from tagged MR image sequences. *IEEE Transactions on Medical Imaging*.

[25] Ryf S, Spiegel MA, Gerber M, Boesiger P (2002). Myocardial tagging with 3D-CSPAMM. *Journal of Magnetic Resonance Imaging*.

[26] Sampath S, Derbyshire JA, Atalar E, Osman NF, Prince JL (2003). Real-time imaging of two-dimensional cardiac strain using a harmonic phase magnetic resonance imaging (HARP-MRI) pulse sequence. *Magnetic Resonance in Medicine*.

[27] Sampath S, Derbyshire JA, Osman NF, Atalar E, Prince JL Real-time imaging of cardiac strain using an ultra-fast HARP sequence.

[28] Spottiswoode BS, Zhong X, Hess AT (2007). Tracking myocardial motion from cine DENSE images using spatiotemporal phase unwrapping and temporal fitting. *IEEE Transactions on Medical Imaging*.

[29] Steenstrup-Pedersen K, Nielsen M, Kerckhove M Computing optic flow by scale-space integration of normal flow.

[30] Suinesiaputra A, Florack LMJ, ter Haar Romeny BM (2003). Multiscale optic flow analysis of MR tagging heart image sequences. *European Journal of Medical Physics*.

[31] Suinesiaputra A, Florack LMJ, Westenberg JJM, ter Haar Romeny BM, Reiber JHC, Lelieveldt BPF Optic flow computation from cardiac MR tagging using a multiscale differential method: a comparative study with velocity-encoded MRI.

[32] Young AA (1999). Model tags: direct three-dimensional tracking of heart wall motion from tagged magnetic resonance images. *Medical Image Analysis*.

[33] Young AA, Axel L (1992). Three-dimensional motion and deformation of the heart wall: estimation with spatial modulation of magnetization: a model-based approach. *Radiology*.

[34] Niessen WJ, Duncan JS, Nielsen M, Florack LMJ, ter Haar Romeny BM, Viergever MA (1997). A multiscale approach to image sequence analysis. *Computer Vision and Image Understanding*.

[35] Gantmacher FR (2001). *The Theory of Matrices*.

[36] Marsden JE, Hughes TJR (1994). *Mathematical Foundations of Elasticity*.

[37] Young AA, Axel L, Dougherty L, Bogen DK, Parenteau CS (1993). Validation of tagging with MR imaging to estimate material deformation. *Radiology*.

[38] Blom J, ter Haar Romeny BM, Bel A, Koenderink JJ (1993). Spatial derivatives and the propagation of noise in Gaussian scale space. *Journal of Visual Communication and Image Representation*.

